# Alcohol-Endocannabinoid Interactions: Implications for Addiction-Related Behavioral Processes

**DOI:** 10.35946/arcr.v42.1.09

**Published:** 2022-05-19

**Authors:** Antonia Serrano, Luis A. Natividad

**Affiliations:** 1 Instituto de Investigación Biomédica de Málaga (IBIMA), Málaga, Spain; 2 Unidad de Gestión Clínica de Salud Mental, Hospital Regional Universitario de Málaga, Málaga, Spain; 3 College of Pharmacy, Division of Pharmacology and Toxicology, University of Texas at Austin, Austin, Texas, USA

**Keywords:** alcohol, dependence, cannabinoids, anxiety, reinforcing, anandamide, 2-arachidonoylglycerol, effects on the brain

## Abstract

**PURPOSE:**

The endogenous cannabinoid system is involved in several physiological functions in the central nervous system including the modulation of brain reward circuitry and emotional homeostasis. Substantial evidence implicates brain endocannabinoid signaling in the processing of drug-induced reward states, wherein repeated exposure besets pathological changes in activity that contribute to the progression of alcohol use disorder. This review provides a narrative summary of recent studies exploring the interaction between alcohol exposure and changes in endocannabinoid signaling that may underlie the development of alcohol use disorder.

**SEARCH METHODS:**

The authors began with an initial search for review articles to assist in the identification of relevant literature. This was followed by separate searches for primary literature and recent studies. The search terms “alcohol/ethanol” and “endocannabinoids” were applied, along with terms that covered specific objectives in reinforcement and addiction behavior. The content was further refined by excluding articles containing a broad focus on psychiatric disorders, polysubstance abuse, non-cannabinoid signaling lipids, and other criteria.

**SEARCH RESULTS:**

The initial search yielded a total of 49 review articles on PubMed, 13 on ScienceDirect, and 17 on Wiley Online, from which the authors garnered information from a total of 16 reviews. In addition to independent searches, this review provides information from a collection of 212 publications, including reviews and original research articles.

**DISCUSSION AND CONCLUSIONS:**

The review discusses the effects of alcohol consumption on brain endocannabinoid signaling, including alcohol-based perturbations in endocannabinoid-mediated synaptic transmission, the modulation of alcohol-related behaviors by manipulating signaling elements of the endocannabinoid system, and the influence of dysregulated endocannabinoid function in promoting withdrawal-induced anxiety-like behavior. Notable emphasis is placed on studies exploring the possible therapeutic relevance of bolstering brain endocannabinoid tone at different stages of alcohol use disorder.

Endogenous cannabinoids, or endocannabinoids (eCBs), are bioactive lipid molecules that modulate signaling activity of several physiological processes involved in pain, appetite, energy balance, stress/anxiety, immune signaling, and learning and memory. Although understanding of the eCB system has grown in complexity since its discovery by Raphael Mechoulam, it is now widely known that eCB systems play an important role in the regulation of brain reward and emotional homeostasis. Given the relevance of these physiological responses in motivated behavior, the hypothesis of the involvement of eCB systems in addiction has been widely investigated.^1^–^3^ Generally, these findings support a role for eCB signaling in mediating the positive reinforcing effects of substances with abuse potential, while repeated drug exposure elicits long-lasting changes aligned with the emergence of negative affective states during abstinence. While these changes ostensibly apply to more than one type of substance with abuse potential, the field has come to understand the strong relation between negative affective states and increased alcohol consumption that facilitates the development of alcohol use disorder (AUD).^4^ Extensive efforts have been made to study the role of eCB systems in alcohol-induced pathologies.^5^,^6^ Highlighted here is recent work exploring the basis of alcohol-eCB interactions in the development of AUD. A brief overview of the molecular constituents involved in eCB synthesis and degradation is followed by a foray of the literature exploring the effect of alcohol consumption on brain eCB signaling. Emphasis is placed on cutting-edge research utilizing genetic and pharmacological approaches to discretely manipulate elements of eCB signaling. This review discusses these findings in terms of the purported roles of the eCBs in synaptic plasticity, stress, and anxiety, and further elucidates the therapeutic relevance of bolstering brain eCB tone in the possible treatment of AUD.

## Search Methods and Results

Searches of the existing literature were primarily conducted on PubMed/PubMed Central. The authors first conducted a broad search of review articles to assist in the identification of primary literature. The terms “alcohol” or “ethanol” and “endocannabinoid” were searched, restricted to the “title/abstract” setting under the “Advanced Search Builder” function. The authors then activated search filters for “Reviews” published within 10 years of June 2021. This search strategy led to the identification of 49 review articles. Similar search strategies in ScienceDirect and Wiley Online Library generated fewer citations (13 and 17, respectively), the majority of which were redundant. To narrow the search more specifically to the goals of the current work, the authors excluded reviews with a broad focus on psychiatric disorders or polysubstance use, fetal drug exposure, non-cannabinoid signaling lipids, phytocannabinoids and other metabolites, as well as eCB/cannabinoid responses outside of the central nervous system. Thus, the authors conducted a thorough reading of 16 reviews.

Separate searches were then conducted to identify primary literature and recent studies using the terms “alcohol/ethanol” and “endocannabinoid” along with general terms covered in each section of the review (e.g., “reward,” “consumption,” “withdrawal/abstinence,” “dependence,” “anxiety,” “FAAH inhibitors,” “MAGL inhibitors”). In some cases, this article refers to reviews and primary literature from major contributors in the field or from the respective laboratories of the authors of this review. All searches were restricted to the English language and generally reflect published work from 1990 to the present, with a few exceptions for foundational work on lipid-alcohol interactions. Most of the studies presented here concern data collected in rodent models. For information on clinical trial testing, the clinicaltrials.gov website was used. This review cites information from a total of 212 publications.

## The eCB System

The eCB system comprises two G-protein coupled receptors, their endogenous lipid ligands, and the enzymes that mediate synthesis and clearance of these molecules. Currently, there are two major types of cannabinoid receptors that are well characterized and cloned: cannabinoid receptor type 1 (CB_1_) and cannabinoid receptor type 2 (CB_2_). CB_1_ receptors are mainly found on presynaptic terminals of neurons in the brain,^7^,^8^ whereas CB_2_ receptors are mostly expressed in immune cells of peripheral tissues,^9^ but are also found in the central nervous system.^10^–^13^ Both receptors are coupled to G_i/o_ protein second messenger systems regulating the amount of cyclic adenosine monophosphate levels in the cell and, by extension, the concentration of intracellular calcium and potassium ions that facilitate synaptic transmission. The relative importance of CB_1_ versus CB_2_ signaling is still under investigation; however, CB_1_ receptors are abundantly found in mesocorticolimbic areas that are important for reward and motivation.^2^,^14^

**Figure 1:**
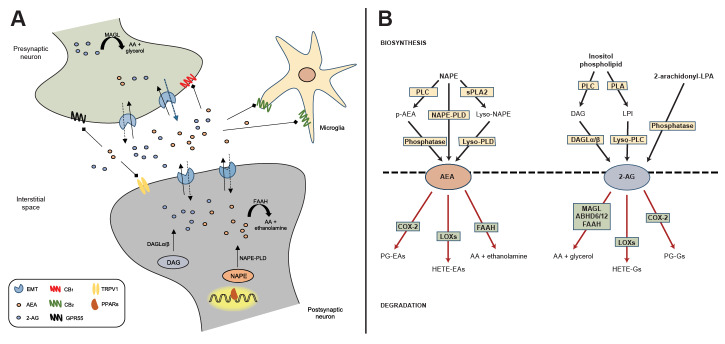
Endocannabinoid signaling and biosynthetic/degradation mechanisms A: Schematic representation of the synaptic organization of the main components of the endocannabinoid system, including established routes of AEA and 2-AG metabolism. B: Metabolic pathways of synthesis and degradation of AEA and 2-AG. See text for details. *Note:* 2-AG, 2-arachidonylglycerol; 2-arachidonoyl-LPA, 2-arachidonoyl-*sn*-glycero-3-phosphate; AA, arachidonic acid; ABHD6/12, alpha/beta-hydrolase domains 6 and 12; AEA, anandamide; CB_1_, cannabinoid receptor type 1; CB_2_, cannabinoid receptor type 2; COX-2, cyclo-oxygenase 2; DAG, diacylglycerol; DAGLα/β, diacylglycerol lipase-alpha/beta; EMT, endocannabinoid membrane transporter; FAAH, fatty acid amide hydrolase; GPR55, G-protein coupled receptor 55; HETE-EAs, hydroxyeicosatetraenoyl-ethanolamides; HETE-Gs, hydroxyeicosatetraenoyl-glycerols; LOXs, lipoxygenases; LPI, lysophosphatidylinositol; lyso-NAPE, lyso-*N*-arachidonoyl-phosphatidylethanolamine; lyso-PLC, lyso-phospholipase C; lyso-PLD, lyso-phospholipase D; MAGL, monoacylglycerol lipase; NAPE, *N*-arachidonoyl-phosphatidylethanolamine; NAPE-PLD, *N*-arachidonoyl-phosphatidylethanolamine-specific phospholipase D; p-AEA, phospho-anandamide; PG-EAs, prostaglandin-ethanolamides; PG-Gs, prostaglandin-glycerols; PLA, phospholipase A; PLC, phospholipase C; PPARs, peroxisome proliferator-activated receptors; sPLA_2_, soluble phospholipase A_2_; TRPV1, transient receptor potential vanilloid type-1.

Currently, the best-studied endogenous ligands of cannabinoid receptors are two arachidonic acid derivatives, *N*-arachidonylethanolamine (anandamide or AEA) and 2-arachidonylglycerol (2-AG). Several other endogenous compounds possess cannabinoid-like properties, although much regarding their pharmacological activity, synthesis, and metabolism remains to be characterized.^15^ AEA and 2-AG activate cannabinoid receptors with a high degree of specificity (see Figure 1A). AEA is a partial agonist of both cannabinoid receptors, with slightly higher affinity for CB_1_ than CB_2_ receptors. On the other hand, 2-AG is a full agonist of both receptors, exhibiting low to moderate affinity for each subtype, and with greater overall potency and efficacy than AEA.^15^,^16^ AEA and 2-AG demonstrate some promiscuity to other receptor systems, including peroxisome proliferator-activated receptors (PPARs) and the orphan G-protein coupled receptors 55 (GPR55) and 119 (GPR119).^17^–^20^ AEA is also known for exerting potent agonist effects on transient receptor potential vanilloid type 1.^21^

Unlike classical neurotransmitters, eCBs are not stored in intracellular compartments but instead are produced “on demand” from membrane lipid precursors in the postsynaptic membrane (see Figure 1B). AEA is produced from the phospholipid precursor *N*-arachidonoyl-phosphatidylethanolamine (NAPE) by a NAPE-specific phospholipase D (NAPE-PLD).^22^ Interestingly, knockdown of NAPE-PLD only moderately depletes AEA signaling pools, suggesting that AEA contains several redundancies in its biosynthesis.^23^ On the other hand, 2-AG is tightly coupled to the production of diacylglycerol from the hydrolysis of an inositol phospholipid by a phospholipase C, which is rapidly converted to 2-AG by two *sn-1*-specific diacylglycerol lipase (DAGL) isoforms (DAGL-alpha and DAGL-beta).^24^,^25^ Emerging research suggests that 2-AG, although widely regarded as the primary synthase, also may be influenced by alternative biosynthetic pathways. One pathway involves the hydrolysis of phosphatidylinositol by a phospholipase A to form a lysophosphatidylinositol, which is hydrolyzed to 2-AG by phospholipase C.^26^ Another alternative pathway is by the dephosphorylation of arachidonic acid-containing lysophosphatidic acid by a phosphatase.^27^

Once released into the synaptic cleft, AEA and 2-AG exert their effects through the retrograde activation of CB_1_ receptors located on presynaptic terminals, followed by rapid termination of signaling via multiple degrading enzymes. In this regard, AEA is primarily degraded by fatty acid amide hydrolase (FAAH) into free arachidonic acid and ethanolamine,^28^ whereas monoacylglycerol lipase (MAGL) is the main enzyme involved in the hydrolysis of 2-AG to produce arachidonic acid and glycerol.^29^ Interestingly, these clearance enzymes are located in different cellular compartments. FAAH is mainly localized to the postsynaptic cell, suggesting a key role for this enzyme in monitoring interstitial AEA concentrations. By contrast, MAGL is mainly found in the presynaptic terminal and contributes to the inactivation of 2-AG near its site of action.^30^ This configuration would suggest that AEA and 2-AG assume different roles in eCB signaling despite the signaling redundancy to cannabinoid receptors. The enzymatic clearance of 2-AG is mostly driven by MAGL,^31^ although other enzymes such as alpha/beta-hydrolase domains 6 and 12 (ABHD6/12)^31^,^32^ and FAAH^33^ have been shown to metabolize 2-AG under certain conditions. AEA and 2-AG also may be oxidized by cyclo-oxygenase 2 and several lipoxygenases^34^,^35^ contributing to the pool of liberated arachidonic acid moieties that can be targeted for eicosanoid production. Overall, these metabolic enzymes play a key role in the production and maintenance of AEA and 2-AG signaling, which portend downstream effects on the regulation of the chemical synapse.

## Neurochemical Role of eCBs in Synaptic Plasticity

The role of the eCB system in synaptic plasticity largely stems from the findings that stimulation of cannabinoid receptors modulates the release of neurotransmitters at excitatory and inhibitory synapses. Further research has characterized the importance of eCB signaling in providing inhibitory control of fast-acting transmitters such as glutamate and gamma-aminobutyric acid (GABA), as well as in modulating activity of other small molecules, such as mesolimbic dopamine.^36^ More generally, eCBs contribute to the shaping of synaptic activity in mesocorticolimbic areas of the brain, which—depending on the strength, frequency, and duration of transmission—can have both immediate and long-lasting consequences on synaptic function.^37^–^42^

Triggering eCB-CB_1_ receptor signaling results in short-term adjustments in neurotransmitter release that modulate activity of the postsynaptic cell via depolarization-induced suppression of excitation or inhibition.^43^–^45^ These transient forms of plasticity typically last a minute or less and are more strongly associated with 2-AG than AEA signaling, although both lipids have been implicated in such responses.^42^ Activation of eCB-CB_1_ receptor signaling can also facilitate more persistent forms of synaptic plasticity, such as long-term depression (LTD). These events vary with the nature of synaptic stimulation but generally persist anywhere from hours to weeks.^42^ The eCB system has long been observed to mediate plasticity in brain regions involved in the etiology of addiction, including the ventral tegmental area, nucleus accumbens (NAc), prefrontal cortex (PFC), hippocampus, amygdala, and dorsal striatum.^1^,^42^,^46^ In this regard, several conceptualizations of addiction theory propose that drug and alcohol exposure result in the disruption of plasticity mechanisms involved in learning and memory, which may contribute further to maladaptations in brain reward circuitry.^47^–^49^

Acute and chronic alcohol exposure disrupts eCB-mediated synaptic plasticity. In this regard, low- to moderate-frequency stimulation of the dorsolateral striatum results in the elevation of eCB levels, which is thought to shift the balance of excitatory and inhibitory regulation of striatal neurons toward long-lasting disinhibition of synaptic output.^50^ Interestingly, acute alcohol exposure impairs this eCB-mediated process and further reduces LTD of medium spiny neurons at inhibitory relative to excitatory synapses.^51^,^52^ The disruption in eCB function is significant given that neural circuits in the dorsal striatum mediate behavioral processes related to reward-guided learning and habitual responding.^53^ In this regard, mice undergoing chronic intermittent alcohol vapor exposure exhibit impaired CB_1_-dependent LTD in the dorsolateral striatum that corresponded with increases in dorsolateral striatal activation and enhanced stimulus-reward learning.^54^ More recently, intermittent alcohol exposure during adolescence conferred long-lasting impairments in CB_1_-dependent LTD in the hippocampus that were associated with disruptions in recognition memory.^55^ These findings suggest that alcohol dysregulates eCB signaling in a manner that fundamentally changes the regulation of the chemical synapse. Impairments in eCB-mediated plasticity likely reflect the loss of an important source of inhibitory constraint of neuronal synapses, leading to pathology in reward-based learning and the modulation of rewarded behavior that influences the progression of AUD.

## Alcohol-Induced Alterations in Brain eCB Levels

One of the more compelling cases for alcohol-eCB interactions regards a series of neuroimaging studies that used positron emission topography to examine CB_1_ receptor binding in humans who smoke cannabis, and then separately in people with AUD.^56^–^58^ Chronic cannabis use produced a striking pattern of CB_1_ receptor downregulation in several (but not all) corticolimbic regions. The results were not surprising given that the psychotropic effects of cannabis are largely mediated by CB_1_ receptor stimulation. Interestingly, patients with AUD showed a similar pattern of dysregulation, though were noted to exhibit decreased binding in all brain regions that were analyzed.^59^,^60^ Moreover, the effects produced by chronic cannabis use returned to normal function after a protracted abstinence period, whereas the disruptions in patients with AUD persisted after 4 weeks of withdrawal from alcohol use. These findings suggest that CB_1_ receptor downregulation is a common neuroadaptation to chronic substance use, although seemingly more extensive under alcohol exposure than with substances that directly interact with CB_1_ receptors. This may suggest that alcohol has potent effects on the mechanisms of CB_1_ receptor expression and function (e.g., signaling transduction, epigenetic changes). Alcohol is also a notable activator of neuroinflammation, which over the course of repeated use may temper the anti-inflammatory responses of exogenous/endogenous cannabinoid signaling.^61^ Moreover, it is possible that alcohol may play a role in altering endogenous mediators of cannabinoid signaling (e.g., eCBs), from which lapses in the recovery of these signaling ligands influence the long-lasting deficits in CB_1_ receptor signaling.

Substantial literature indicates that brain eCB content is altered by substances with abuse potential. In this regard, alcohol alters AEA and 2-AG content in the brain, and chronic alcohol exposure generally leads to impairments in eCB signaling mechanisms. Early in vitro studies demonstrated that chronic alcohol exposure increases both AEA and 2-AG formation in human neuroblastoma cells and primary cultures of rodent cerebellar granule neurons.^62^–^64^ Subsequent studies have evaluated the effects of alcohol exposure on brain eCB levels and reported differential effects.^65^ Currently, it is difficult to draw a firm consensus of these data given the plethora of responses induced by alcohol administration, which may include—in addition to sample preparation, brain-region specificity, and methodological differences—the differential mobilization of AEA and 2-AG. Highlighted below are some of these findings, summarized in Table 1.

Chronic alcohol exposure has been shown to increase AEA content in the limbic forebrain of rodents, whereas withdrawal decreased AEA in these brain regions.^66^–^69^ This increase in AEA is consistent with the reduction in FAAH activity following chronic alcohol exposure.^66^ By contrast, protracted (but not acute) withdrawal increased AEA content in the rat hippocampus.^70^ Short-term alcohol exposure also has been reported to decrease AEA content in several brain regions including the amygdala, hypothalamus, and caudate putamen.^71^ Regarding 2-AG, several studies describe both increases and decreases in striatal 2-AG content after chronic alcohol exposure.^67^,^68^,^72^ Moreover, acute and protracted withdrawal from chronic intermittent alcohol exposure was observed to increase 2-AG content in the rat hippocampus.^70^ In the PFC, acute alcohol exposure was associated with decreases in 2-AG content,^71^ whereas voluntary consumption in genetically selected rats that were bred for high alcohol preference was shown to increase 2-AG in this region.^69^ Drinking behavior in Sardinian alcohol-preferring (sP) rats also was associated with increases in striatal 2-AG content that were most evident during the acquisition and maintenance phases.^72^ These varied responses between studies are likely influenced by methodological differences in the procedure employed to quantify eCB tissue content,^73^ as well as by other experimental factors including the selection of rodent model, rat strain, duration and amount of alcohol exposure, and timepoints of withdrawal assessment. Emerging research also suggests the possibility of sex differences in alcohol-eCB interactions that may be specific to ovarian hormones.^69^,^74^

As opposed to bulk eCB tissue levels, some laboratories have utilized in vivo microdialysis approaches to estimate changes in eCB levels in flux.^73^ These studies likewise have reported region-specific effects in alcohol administration, as well as the influence of several factors involved in the administration, dose, contingency, and prior history of alcohol exposure.^75^,^76^ Seminal work from Larry Parsons’ laboratory demonstrated that operant alcohol self-administration increased interstitial levels of 2-AG in the NAc without altering dialysate levels in the medial PFC.^77^,^78^ Systemic administration of moderate doses of alcohol also increased 2-AG levels in a similar manner in alcohol-naïve rats, and this effect was potentiated in alcohol dependence.^76^ More recently, the authors observed that alcohol dependence resulted in the reduction of baseline 2-AG levels in the central nucleus of the amygdala (CeA), conferring a blunting of alcohol’s mobilizing responses in this region.^79^ Regarding AEA, alcohol self-administration did not differentially alter interstitial levels of AEA across several brain regions.^76^,^77^,^79^ Interestingly, noncontingent alcohol administration reduced AEA in the NAc, whereas higher doses produced a milder increase in dialysate levels.^75^,^76^,^80^ Alcohol dependence also did not appear to drastically alter baseline AEA levels in the CeA.^79^

Overall, it is clear that alcohol administration alters eCB responsivity, albeit in a manner that is dependent on several factors of exposure. What is less clear, however, is the manner in which alcohol may be mobilizing these responses, let alone with any given specificity to eCB signaling. Previous studies have shown that alcohol possesses cell membrane-disrupting properties that build tolerance over the course of repeated exposure. This resistance is conferred through the alteration of lipid membrane composition that includes changes in important glycerophospholipids such as phosphatidylinositol, cardiolipin, and several classes of amino glycerophospholipids (e.g., phosphatidylcholine, phosphatidylserine, phosphatidylethanolamine).^81^,^82^ The changes in phospholipid content vary with the nature of alcohol-induced perturbation, demonstrating higher depletion effects under intermittent versus continuous exposure conditions.^83^ Acute withdrawal also has membrane-disordering consequences in different cellular compartments that were previously acclimated to the presence of alcohol.^81^ Collectively, these findings suggest that alcohol exposure and withdrawal perturb the integrity of the cellular lipid bilayer, which may be important for determining the source of glycerophospholipid content available for eCB synthesis. In this regard, depletions in inositol phospholipid content would seemingly have profound implications in the ability to mobilize 2-AG synthesis relative to AEA systems that contain biosynthetic redundancies for recuperating losses.

## The Influence of eCB Systems on Alcohol-Related Behaviors

Given the precedence for alcohol-eCB dysregulation, there are several avenues for which one might explore the role of eCB systems in addiction behavior. Although many studies point to the influence of CB_1_ receptors, recent advancements have made it possible to discretely manipulate eCB signaling elements. Highlighted below are some of these investigations that underscore the involvement of eCB systems in alcohol-related behaviors. Table 2 provides a summary of the main findings for cannabinoid receptors.

### CB_1_ Receptors

The consensus of preclinical work demonstrates that activation of CB_1_ receptors has a facilitatory effect on the motivation and consumption of alcohol. For example, systemic administration of the synthetic CB_1_ receptor agonists WIN 55,212-2 and CP 55,940 both increased spontaneous drinking in sP rats and mice.^84^–^87^ These synthetic agonists also increased operant responding for alcohol in Alko alcohol rats and Indiana P rats, as well as in non-selected Wistar rats.^88^–^90^ The facilitatory effect on alcohol consumption likely involves the activation of mesolimbic CB_1_ receptors, given that both systemic and intracranial infusions of WIN 55,212-2 into the posterior ventral tegmental area increased binge-like alcohol intake.^91^ Additional studies have shown that WIN 55,212-2 administration increased the magnitude of excessive drinking elicited by the alcohol deprivation effect.^92^,^93^ Conversely, the pharmacological blockade of CB_1_ receptors by the CB_1_ antagonist/inverse agonist SR141716A (rimonabant) decreased alcohol consumption in non-selected and alcohol-preferring rats and mice.^86^,^94^–^98^ This decrease was observed in both dependent and non-dependent rodent models^98^,^99^ and was further associated with reduced motivation for alcohol.^97^,^100^ SR141716A also reduced the magnitude of alcohol deprivation effect responses in alcohol-preferring rats^72^,^90^,^101^ and treatment with other selective CB_1_ antagonists/inverse agonists recapitulated many of these same effects.^102^–^105^ Consistent with this, the genetic ablation of CB_1_ receptors in mice attenuated alcohol preference and intake,^86^,^106^–^108^ diminished the influence of SR141716A pharmacology,^108^ and reduced preference for environments previously paired with alcohol reward (e.g., conditioned place preference [CPP]).^109^ This likely has some bearing with the modulation of mesolimbic dopamine given that alcohol’s ability to increase NAc dopamine release was compromised in CB_1_ receptor knockout mice.^106^

Overall, these findings demonstrate that while activation of CB_1_ receptors promotes alcohol consumption, the pharmacological blockade or genetic deletion of these receptors has the opposite effect.^110^ The results underscore the importance of CB_1_ receptors in alcohol-related behaviors, although there is less clarity regarding the signaling substrates that mediate these responses. In this regard, the authors’ recent work demonstrated that SR141716A infused directly into the NAc shell decreased alcohol self-administration and this tempering response was recapitulated with the exogenous administration of 2-AG, but not AEA into this region.^111^ The findings suggest the possibility of 2-AG–CB_1_ signaling being an important mediator in the reinforcing effects of alcohol, although the possibility of non-cannabinoid signaling pathways has not yet been ruled out. These findings have translational relevance in the clinic given that polymorphisms of the *Cnr1* gene that encodes for CB_1_ receptors were associated with symptoms of AUD.^112^

### CB_2_ Receptors

Although numerous findings corroborate the involvement of CB_1_ receptors in alcohol-related pathology, the possible role of CB_2_ receptors remains somewhat controversial. Brain CB_2_ signaling is typically engaged under marked conditions of neuroinflammation and tissue trauma,^113^ and the extent to which drugs of abuse may elicit such phenotypes is currently under investigation. That being stated, sub-chronic treatment with the CB_2_ receptor agonist JWH-015 was reported to increase chronic stress-induced alcohol consumption, whereas similar protocols with the CB_2_ receptor antagonist AM630 prevented alcohol preference.^114^ The naturally available full-agonist of CB_2_ receptors, beta-caryophyllene, had dissimilar effects and instead decreased preference and consumption as well as inhibited the expression of alcohol-induced CPP.^115^ Studies using the selective CB_2_ agonist JWH-133 also reported contradictory findings, in some cases showing the attenuation of alcohol-induced CPP and operant self-administration,^116^,^117^ and in others having no effect on these behaviors.^118^,^119^ The varied responses may be due to experimental factors such as the method and duration of alcohol exposure, the mouse strain utilized, or the dose of agonist administered prior to testing.

The blockade of CB_2_ receptors has somewhat more consistent effects that align with increased reinforcement and motivation for alcohol. For example, repeated administration of the antagonist AM630 increased operant alcohol self-administration in mice,^117^ although others reported no effects on alcohol intake or alcohol-induced CPP.^114^,^118^ Behavioral phenotyping in CB_2_ receptor knockout mice has shown that these animals exhibit increased alcohol preference and consumption, elicit more physical signs of alcohol dependence,^120^ and express higher alcohol-induced CPP than wild-type controls.^118^,^120^ By contrast, knockout mice of a different strain did not exhibit significant differences in limited-access drinking,^118^,^121^ but interestingly showed an increase in alcohol intake under forced alcohol exposure and group-housing conditions. These data suggest the possibility that CB_2_ receptors may tie into complex interactions of alcohol and stress that is facilitated by the social environment.^121^ Targeting the deletion of CB_2_ receptors in dopamine neurons also reduced alcohol consumption and mitigated the expression of alcohol-induced CPP in DAT-*Cnr2* conditional knockout mice.^116^ These findings may bear some translational relevance in the clinical field given that polymorphisms in the CB_2_ receptor gene (*Cnr2*) were associated with AUD in Japanese populations.^114^

### Inhibition of eCB Clearance

The modulation of cannabinoid receptors provides a strong basis for alcohol-eCB interactions; however, the recent development of novel pharmacological and genetic tools that prevent the clearance of eCBs provides a means to discern the roles of these lipids in alcohol-induced behavior. Table 3 summarizes the information below.

### FAAH Inhibition

The inhibition or genetic deletion of the clearance enzyme FAAH results in an increase in AEA levels as well as other acylethanolamines such as oleoylethanolamine and palmitoylethanolamine.^122^ Growing evidence suggests that impairment of FAAH may prime sensitivity to the reinforcing effects of alcohol and attenuate the negative consequences of excessive drinking. For example, acute administration of the FAAH inhibitor URB597 in mice increased alcohol preference and consumption, while also reducing sensitivity to the motor-impairing responses of intoxication.^85^,^123^,^124^ Similar effects were observed in the genetic deletion of FAAH in mice,^85^,^123^,^124^ that among other attributes promoted the quick recovery of alcohol-induced motor discoordination. The pharmacological effects of URB597 were further abrogated in CB_1_ receptor and FAAH knockout mice, and behavioral sensitization to repeated alcohol administration was diminished in these mouse lines.^125^ Contrary to the findings in mice, URB597 administration did not alter voluntary drinking in alcohol-preferring rats or operant responding in non-selected Wistar rats.^126^,^127^ The authors observed similar findings with the administration of the selective FAAH inhibitor PF-3845 in both dependent and nondependent rats.^79^ Thus, although FAAH inhibition may differentially alter alcohol-related behaviors in mice, it is less clear whether similar phenotypes exist in rat models. Alternatively, several studies have demonstrated that inhibiting FAAH more discretely within corticolimbic areas of the brain resulted in observable phenotypes. For example, the local administration of URB597 into the PFC of non-selected rats facilitated operant alcohol self-administration, and this effect was consistent with observations of decreased FAAH expression and activity in the PFC of alcohol-preferring Alko alcohol rats.^126^ By contrast, infusions of URB597 into the CeA or the basolateral amygdala reduced alcohol self-administration in Marchigian Sardinian alcohol-preferring (msP) rats, while having no effect in non-selected Wistar rats.^128^ The msP rat line has been previously shown to exhibit elevated FAAH activity in amygdalar brain regions,^129^ suggesting that facilitation or inhibition of alcohol drinking may largely depend on the status of AEA signaling in these corticolimbic regions. Thus, peripheral administration of an FAAH inhibitor is likely to offset the region-specific differences in AEA clearance, not surprisingly culminating in a null response on alcohol drinking.

Recent work has explored the contribution of FAAH mechanisms in driving alcohol-seeking behavior. Consistent with the studies above, FAAH inhibition in mice reduced reinstatement-induced drinking in a CB_1_-dependent manner.^130^ In rats, the peripheral administration of URB597 did not facilitate operant responding in an alcohol reinstatement model,^127^ nor did it moderate alcohol reinstatement driven by pharmacological stressors. However, the local administration of URB597 into the lateral habenula reduced voluntary consumption and preference in alcohol-dependent rats^131^ and reduced alcohol-seeking behavior; these effects were effectively reversed by co-administration of rimonabant. The lateral habenula has garnered recent interest in the addiction field given its role in mediating negative valence information that may contribute to the negative symptoms of withdrawal.^132^ Dysregulation of FAAH is also observed in the clinic, given that a missense mutation in FAAH (e.g., the C385A polymorphism) was associated with heightened prevalence of AUD,^133^,^134^ and increased risk of developing alcohol problems in young people.^135^

### Inhibition of eCB Transport

Currently, the mechanisms mediating fatty acid sequestration and membrane transport of the eCBs are unclear, although a few studies have elucidated the effects of an active metabolite of acetaminophen (i.e., AM404) in modulating alcohol-related behaviors. AM404 is thought to prevent the uptake of AEA and 2-AG, in effect prolonging synaptic signaling of these lipids.^136^–^138^ In mice, AM404 reduced alcohol-seeking behavior and consumption.^139^ Similarly, this compound reduced alcohol self-administration in Wistar rats at doses that did not alter saccharin self-administration, though no effects were observed in cue- or stress-induced reinstatement models.^140^

### MAGL Inhibition

Although many studies have characterized the role of AEA/FAAH signaling systems on alcohol-related behaviors, the possible relevance of 2-AG/MAGL is only beginning to be explored with the development of selective and efficacious tools for inhibiting MAGL. In this regard, the authors have shown that local administration of the selective MAGL inhibitor URB602 into the NAc shell reduces operant alcohol self-administration in rats.^111^ In addition, acute administration of the inhibitor MJN110 reduced operant self-administration in alcohol-dependent rats, and in separate studies reduced voluntary drinking in dependent mice using the inhibitor JZL184.^79^ Consistent with these findings, increased MAGL activity was observed in the lateral habenula of dependent rats, and intracranial infusions of JZL184 reduced alcohol consumption in a CB_1_-dependent manner.^131^ Thus, as opposed to the varied responses obtained with systemic FAAH inhibitors, the dysregulation of 2-AG/MAGL signaling in dependence appears to be a pervasive or stable phenotype. That stated, a more time-dependent profiling of the changes induced by chronic alcohol exposure and withdrawal is warranted and should provide a better means of discerning the therapeutic potential of FAAH and MAGL inhibitors in AUD.

## Endocannabinoids and Withdrawal-Related Anxiety

Repeated cycles of alcohol intoxication and withdrawal induce neuroadaptations that alter the motivational mechanisms involved in compulsive alcohol seeking and drinking.^141^ Although initial use is motivated by the hedonic effects of alcohol, prolonged exposure results in the blunting of brain reward pathways that are overcome pharmacologically by escalating alcohol intake. At the same time, opponent processes involved in the remediation of mood states gain traction and contribute to the expression of negative affect during periods of alcohol abstinence. This rise in sensitivity marks a transition point where alcohol use becomes an effective means of alleviating negative behavioral states, thus creating a psychological tangent for the progression of AUD. Namely, withdrawal-induced increases in negative affective states (e.g., hyperkatifeia^4^) arise from the combination of stress signaling factors that activate areas of the extended amygdala (e.g., corticotropin-releasing factor [CRF]) and diminished performance of the mechanisms that constrain these responses through so-called “anti-stress” functions.^142^ Growing evidence implicates the eCB system as a prevailing mechanism in the regulation of stress signaling,^112^,^143^,^144^ and by extension of this basic function, reflects the loss of a critical “anti-stress” mechanism in AUD.^145^ Highlighted below is some of the research supporting the framework for dysregulated eCB signaling in the manifest of negative affective behavior associated with alcohol withdrawal.

Substantial evidence shows that eCB systems play a key role in the modulation of stress signaling, wherein disruptions of eCB signaling can facilitate anxiety-like states.^146^ CB_1_ receptors are expressed in high or moderate densities across many regions involved in the expression of anxiety, including the CeA, basolateral amygdala, PFC, ventral hippocampus, and bed nucleus of the stria terminalis.^8^,^147^,^148^ As with the findings observed in human subjects with AUD, the downregulation of CB_1_ receptors appears to be an important attribute of mood affective disorders, at least within subcortical regions that are posited to interact more frequently with upstream hormonal regulators.^149^

Cannabis use in humans is known to alter anxiety-like states in a dose-dependent manner.^150^,^151^ For example, the acute administration of Delta^9^-tetrahydrocannabinol (THC) produces anxiolytic responses at low doses,^152^–^155^ but elicits anxiogenic effects with progressively higher doses.^152^,^156^,^157^ Synthetic agonists of CB_1_ receptors display similar propensities in rodents that are abrogated with a CB_1_ receptor antagonist.^158^,^159^ Interestingly, not all agonists modulate anxiety-like behavior in the same manner and instead display complex interactions with the testing environment. Indeed, low doses of the agonist HU-210 were observed to contain anxiolytic-like effects in a model of defensive withdrawal behavior when tested in novel environments, whereas similar doses under habituated settings produced anxiogenic-like responses.^160^ Given that CB_1_ receptors are located on the terminals of glutamatergic and GABAergic neurons,^161^ it is hypothesized that the regulation of anxiety-like behavior may relate more specifically to the subpopulation of neurons influenced by CB_1_ receptor activation. In this regard, studies using conditional mutant mice lacking CB_1_ receptors within specific neurons reported that low-dose activation of CB_1_ receptors on glutamatergic neurons was associated with anxiolytic-like responses, whereas high doses of agonist that disrupted GABAergic signaling were anxiogenic.^162^–^164^

There is now considerable evidence demonstrating that elevations in eCB levels (via the inhibition of clearance mechanisms) modulate anxiety-like behavior without inducing the same biphasic responses obtained with CB_1_ receptor agonists. For example, the indirect stimulation of AEA signaling by FAAH inhibitors reduced the expression of anxiety-like behaviors in rodents but did so specifically under stressful or aversive conditions.^129^,^165^–^168^ Similar effects were obtained in FAAH knockout mice.^169^,^170^ In addition to AEA/FAAH signaling, there is evidence supporting the role of 2-AG/MAGL in the regulation of anxiety-like behavior. In this regard, the MAGL inhibitor JZL184 produced anxiolytic-like effects in rodents mainly under heightened stress conditions (e.g., brightly lit environments, following restraint stress).^165^,^168^,^171^–^174^ Unlike the anxiolytic effects of FAAH inhibitors that are strongly associated with CB_1_ receptor signaling,^144^ both CB_1_ and CB_2_ receptors have been implicated in the anxiety-reducing properties of MAGL inhibitors;^173^–^176^ to date, however, the preponderance of evidence suggests a CB_1_ receptor contingency.

The authors’ recent work with msP rats provides collective evidence of the strong relation between dysregulated AEA/FAAH signaling and innate symptoms of anxiety.^129^ In this regard, msP rats are genetically selected for increased alcohol preference and consumption, as well as for the heightened expression of anxiety-like behavior.^177^ Accordingly, the authors observed that msP rats displayed a sensitized stress response in the CeA and provided evidence of diminished AEA neurotransmission driven by increased clearance of this lipid by FAAH. Inhibition of FAAH with PF-3845 rescued the msP phenotype in several models of anxiety-like behavior, likely by restoring the integrity of stress-gating control in the CeA. Subsequent work demonstrated that local administration of the inhibitor URB597 into the CeA reversed the anxiety-producing effects of restraint stress, whereas no effects were observed in non-selected Wistar rats.^128^ Consistent with this, the authors also have examined the effects of FAAH and MAGL inhibitors on withdrawal-induced anxiety-like behaviors in rodents and found that both inhibitors were effective in reducing these responses.^79^ Given the tempered effects of systemic FAAH inhibitors in alcohol drinking behavior, it is tempting to suggest that AEA and 2-AG may be regulating different components of the addiction process, the former being more attuned to the regulation of basal anxiety levels and the latter being consequential of alcohol-induced perturbations. How this may fit into a gain- or loss-of-function model that can inform the therapeutic relevance of eCB clearance inhibitors remains to be elucidated. Additionally, the interactive role of eCB systems with stress-inducing factors such as CRF and other stress-constraining mechanisms such as cortisol/corticosterone is not well understood. In this regard, previous work suggests that neuroadaptations involving CRF-driven stimulation of FAAH coincide with the depletion of AEA-mediated constraint of the amygdala,^129^,^178^ whereas the delayed and blunted release of corticosterone in msP rats^179^ may present a challenge in mounting 2-AG remediation.^180^

Unlike the selective FAAH or MAGL inhibitors, the increase of AEA and 2-AG levels with the dual eCB clearance inhibitor JZL195 has little effect on reducing anxiety-like behavior and instead appears to have anxiogenic-like properties.^165^,^181^ Recently, the authors observed evidence of an anxiolytic-like effect with high doses of JZL195 on the elevated plus maze, but similar treatments had no effect in the light/dark box assay.^168^ Moreover, treatment with the MAGL inhibitor JZL184 in FAAH knockout mice, mimicking the putative inhibitor properties of JZL195, did not produce any effects on anxiety-like behaviors. It should be borne in mind that dual FAAH/MAGL inhibition produced cannabimimetic effects^182^ and prolonged changes in 2-AG signaling (via MAGL inhibitor treatment in FAAH knockout mice) that were associated with cannabinoid receptor dysregulation, tolerance to antinociception, and increased sensitivity to rimonabant-precipitated withdrawal behavior.^183^ The potential role of dual FAAH/MAGL inhibition has not been thoroughly examined in alcohol-dependent rodents, but has been shown to contain neurogenesis-suppressing effects in the dentate gyrus in the same manner as the combined treatment of acute alcohol with a CB_1_ agonist.^184^

Other studies have observed that the loss of 2-AG signaling through the genetic or pharmacological inhibition of synthase mechanisms is associated with anxiogenic-like responses. For example, DAGL-alpha knockout mice exhibit increased anxiety-like behaviors relative to their wild-type littermates,^185^,^186^ and these effects were reversed by the administration of JZL184.^185^ In the same regard, the DAGL inhibitor DO34 produced anxiogenic-like effects,^187^ although the extent to which prior stress conditions may differentially influence the expression of anxiety-like behavior remains to be elucidated. Given evidence of alcohol’s mobilizing properties of 2-AG signaling, it is possible that DAGL inhibition may serve as a novel therapeutic for the treatment of AUD. Indeed, recent studies are providing insight into the possible therapeutic relevance of DAGL inhibition in reducing alcohol consumption without precipitating negative affective behaviors associated with chronic alcohol exposure and withdrawal.^188^

In addition to preclinical work, clinical studies are underway to evaluate the therapeutic efficacy of eCB enzyme inhibitor treatment in humans. Currently, there is more information on pharmacological inhibitors of FAAH given that selective inhibitors of MAGL have been characterized only recently.^189^ The FAAH inhibitor PF-04457845 has entered Phase 2 clinical testing for the treatment or study of several conditions including chronic pain, fear response, Tourette’s syndrome, and cannabis use disorder. PF-04457845 was found to be safe, well tolerated, and—although showing negligible effects for analgesia—successful in facilitating fear extinction behavior in healthy individuals.^190^,^191^ More recently, PF-04457845 was reported to reduce withdrawal symptoms and cannabis use in patients with cannabis use disorder.^192^ Other FAAH inhibitors, such as JNJ-42165279 and ASP3652, also were found to be safe and well tolerated; although confirming the lack of efficacy for chronic pain, these FAAH inhibitors displayed anxiolytic effects in people with social anxiety disorders.^193^–^197^ By contrast, the FAAH inhibitor BIA 10-2474 caused widespread concern when high doses of this drug induced neurotoxic effects in healthy individuals, ending in the death of one volunteer.^198^ It was later reported that BIA 10-2474 displayed substantial “off-targets” that were unique to this drug and likely responsible for inducing metabolic dysregulation and cellular death.^199^ Although future studies should continue to ascertain the safety profile of FAAH inhibitors, the positive responses observed in people with cannabis use disorder bode well for substance abuse treatment. Together with the recent development of selective MAGL inhibitors (ABX-1431) in clinical trial testing,^200^ serine hydrolase inhibitors represent a possible treatment avenue for restoring dysfunctional cannabinoid signaling in people with AUD.

## Conclusion and Future Directions

Despite some inconsistencies in the literature, a preponderance of evidence suggests that alcohol exposure alters brain eCB signaling. Findings from the Parsons’ laboratory demonstrated that acute alcohol self-administration elicits increases in eCB release that are tempered over repeated exposure;^76^,^79^ however, readers are referred to the Alcohol-Induced Alterations in Brain eCB Levels section of this review for noteworthy distinctions. In addition, the method of alcohol exposure plays a marked role in the subsequent analysis of abstinence-related effects.^201^,^202^ That stated, chronic alcohol exposure is generally associated with the disruption of eCB clearance mechanisms, impaired eCB-mediated forms of synaptic plasticity, and the downregulation of cannabinoid receptor function. The dysregulation of eCB signaling may be relevant given that eCBs play a prominent role in the maintenance of affective states and the constraint of stress responses, both of which serve as provocateurs of continued use and relapse. The remediation of eCB signaling remains an important goal for the possible treatment of AUD; however, this is unlikely to be achieved through the exogenous manipulation of CB_1_ receptors that are fraught with concerns.^202^–^205^ Accordingly, eCB clearance therapeutics may present an alternative pathway for restoring dysfunctional signaling elements, although further research is needed to better understand the consequence of eCB augmentation in dependence states across other relevant variables, including sex, brain regions, environment, emotional valence, pre-existing conditions, and neurohormones.^206^

Understanding of eCB signaling has greatly evolved since the discovery of eCBs nearly 30 years ago. This was fueled by technological advancements in the isolation, detection, and sequencing of the two primary eCBs, as well as the crystallization of biosynthetic enzymes and receptor systems that enable them. Cutting-edge technology continues to be an important driver in the field for the identification of novel molecular species and distinctions in eCB function. For example, mass spectrometry analysis can be broadly applied to investigate the brain lipidome, from which metabolic products of eCB degradation are utilized by downstream signaling pathways (e.g., eicosanoids) to mediate neuroinflammation.^207^ This is coupled closely to the advancements of novel pharmacological tools such as DO34 and the NAPE-PLD inhibitor LEI-401^208^ that will allow us to manipulate AEA and 2-AG signaling with great precision and selectivity. Moreover, the spatiotemporal resolution of such changes is fundamental to the understanding of eCB function and may provide insight on the purpose of having multiple endogenous ligands of cannabinoid receptors. Although traditionally studied with in vivo microdialysis, the recent development of G-protein coupled receptor activation-based eCB sensors offers subsecond resolution kinetics and robust fluorescence-based detection in awake-behaving rodents.^209^ Finally, the development of novel positron-emission topography tracers such as [^11^C]MK-3168^210^ and [^18^F]T-401^211^ will allow the direct assessment of FAAH and MAGL activity under a number of planned clinical studies, including in people with AUD. Taken all together, emerging research appears to be on the precipice of divulging new information about the eCB system. The combination of selective pharmacology and in vivo capture methods remains an important endeavor in this research for answering fundamental questions of eCB function, its relation to stress and anxiety, and its higher-order influence in complex psychopathologies such as AUD and addiction.

## Figures and Tables

**Table 1:** Summary of Alcohol-Induced Alterations in Brain eCB Levels

Type of Study (cell/species)	Alcohol Exposure	Effects	Brain Region

In vitro (human neuroblastoma cells)	Chronic alcohol	▲AEA	N/A

In vitro (rodent cerebellar granule neurons)	Chronic alcohol	▲AEA ▲2-AG	N/A

Ex vivo tissue content (male Swiss Webster mice)	Chronic vapor inhalation	▲AEA	Cortex

	Acute withdrawal	▼AEA	Cortex

Ex vivo tissue content (male Wistar rats)	Chronic liquid diet	▼AEA ▼2-AG	Midbrain

		▲AEA	Limbic forebrain

	Acute withdrawal	▼AEA	Limbic forebrain

Ex vivo tissue content (male Sprague-Dawley rats)	Acute withdrawal	►AEA ▲2-AG	Hippocampus

	Long-term withdrawal	▲AEA ▲2-AG	

	Short-term alcohol exposure (liquid diet for 24h)	▼AEA	Hypothalamus Amygdala Caudate putamen

		▼2-AG	PFC

Ex vivo tissue content (female and male alcohol-preferring AA rats)	Long-term alcohol consumption in female: Before drinking session	▲AEA	PFC NAc CPu
		▲2-AG	CPu Amygdala Hippocampus
	After drinking session	▼AEA ▲2-AG	PFC CPu Amygdala Hippocampus PFC

	Long-term alcohol consumption in male: Before drinking session	►AEA ►2-AG	PFC NAc CPu Amygdala Hippocampus
	After drinking session	▲AEA	NAc CPu

Ex vivo tissue content (male sP rats)	Long-term voluntary alcohol consumption	▲2-AG	Striatum

Ex vivo tissue content (male and female Wistar rats)	Acute withdrawal male	▼AEA ▼2-AG	BLA vmPFC

	Acute withdrawal female	▼AEA	vmPFC

In vivo microdialysis (male Wistar rats)	Alcohol self-administration	▲2-AG ►AEA ►2-AG	NAc mPFC

In vivo microdialysis (male Wistar rats)	Acute alcohol administration in naïve rats (low doses) Acute alcohol administration in naïve rats (high doses)	▲2-AG ▼AEA ▲AEA	NAc

	Acute alcohol administration in alcohol-dependent rats	▲▲2-AG ► AEA	NAc

In vivo microdialysis (male Wistar rats)	Chronic alcohol exposure	▼2-AG ► AEA	CeA CeA / NAc

*Note:* ▲, increase;▼, decrease; ►, no effect; 2-AG, 2-arachidonylglycerol; AA rats, Alko alcohol rats; AEA, anandamide; BLA, basolateral amygdala; CeA, nucleus of the central amygdala; CPu, caudate putamen; mPFC, medial prefrontal cortex; NAc, nucleus accumbens; PFC, prefrontal cortex; sP rats, Sardinian alcohol-preferring rats; vmPFC, ventromedial prefrontal cortex.

**Table 2:** Summary of CB Receptor Influence on Alcohol-Related Behaviors

CB Receptor Manipulation	Effects

**CB**_1_ **receptor agonists**	▲spontaneous drinking in alcohol-preferring rodents ▲alcohol SA in rats ▲binge-like alcohol intake in mice ▲alcohol-seeking behavior

**CB**_1_ **receptor antagonists**	
systemic administration	▼alcohol preference ▼alcohol consumption in rodents ▼alcohol-seeking-behavior
localized infusions: intra-NAc	▼alcohol SA
intra-VTA	▼alcohol SA
intra-mPFC	►alcohol SA in normal rats
intra-PFC	▼alcohol SA in alcohol-preferring rats

**CB**_1_ **receptor knockout mice**	▼alcohol preference ▼alcohol consumption in rodents ▼CPP ▼alcohol-induced NAc dopamine

**CB**_2_ **receptor agonists**	▲alcohol consumption in stressed mice ▼CPP / ►CPP ▼alcohol preference ▼alcohol consumption / ►alcohol consumption ▼alcohol SA

**CB**_2_ **receptor antagonists**	▲alcohol SA

**CB**_2_ **receptor knockout mice**	▲alcohol consumption ▲alcohol preference ▲physical signs of withdrawal ▲CPP

*Note:* ▲, increase;▼, decrease; ►, no effect; CB, cannabinoid; CB_1_ receptor, cannabinoid receptor type 1; CB_2_ receptor, cannabinoid receptor type 2; CPP, conditioned place preference; mPFC, medial prefrontal cortex; NAc, nucleus accumbens; PFC, prefrontal cortex; SA, self-administration; VTA, ventral tegmental area.

**Table 3:** Summary of eCB Clearance Inhibition Influence on Alcohol-Related Behaviors

eCB Clearance Manipulation	Effects

**FAAH inhibitors**	
systemic administration	▲alcohol preference in mice, but not rats ▲alcohol consumption in mice, but not rats ▼sensitivity to alcohol intoxication
localized infusions: intra-PFC	▲alcohol SA in rats
intra-amygdala	▼alcohol SA in msP rats ►alcohol SA in Wistar rats
intra-LHb	▼alcohol preference in alcohol-dependent rats ▼alcohol consumption in alcohol-dependent rats ▼alcohol-seeking behavior

**FAAH knockout mice**	▲alcohol preference ▲alcohol consumption ▼sensitivity to alcohol intoxication

**eCB transport inhibitor**	▼alcohol seeking ▼alcohol consumption ▼alcohol SA in rats

**MAGL inhibitors**	
systemic administration	▼alcohol intake in alcohol-dependent rodents ►alcohol intake in non–alcohol-dependent rodents
localized infusions: intra-NAc shell	▼alcohol SA in rats
intra-LHb	▼alcohol consumption in alcohol-dependent rats ►alcohol consumption in non–alcohol-dependent rats

*Note:* ▲, increase;▼, decrease; ►, no effect; eCB, endocannabinoid; FAAH, fatty acid amide hydrolase; LHb, lateral habenula; MAGL, monoacylglycerol lipase; msP rats, Marchigian Sardinian alcohol-preferring rats; NAc, nucleus accumbens; PFC, prefrontal cortex; SA, self-administration.
